# Interleukin-6 and activin A are independently associated with cardiovascular events and mortality in type 2 diabetes: the prospective Asker and Bærum Cardiovascular Diabetes (ABCD) cohort study

**DOI:** 10.1186/1475-2840-12-126

**Published:** 2013-08-30

**Authors:** Anne Pernille Ofstad, Lars Gullestad, Elsa Orvik, Svend Aakhus, Knut Endresen, Thor Ueland, Pål Aukrust, Morten W Fagerland, Kåre I Birkeland, Odd Erik Johansen

**Affiliations:** 1 Department of Medical Research, Bærum Hospital, Vestre Viken Hospital Trust, N-1309 Rud, Bærum, Norway; 2 Department of Cardiology, Oslo University Hospital Rikshospitalet, Oslo, Norway; 3 K.G.Jebsen Cardiac Research Centre and Center for Heart Failure Research, Faculty of Medicine, University of Oslo, Oslo, Norway; 4 Research Institute of Internal Medicine, Oslo University Hospital Rikshospitalet, Oslo, Norway; 5 Section of Clinical Immunology and Infectious Diseases, Oslo University Hospital Rikshospitalet, Oslo, Norway; 6 Institute of Clinical Medicine, University of Oslo, Oslo, Norway; 7 Unit of Biostatistics and Epidemiology, Oslo University Hospital, Oslo, Norway; 8 Department of Endocrinology, Obesity and Preventive medicine, Oslo University Hospital Aker, Oslo, Norway

**Keywords:** Type 2 diabetes, Cardiovascular event, MACE, Inflammatory marker, Cardiovascular risk, Risk prediction

## Abstract

**Background:**

Novel and robust cardiovascular (CV) markers are needed to improve CV morbidity and mortality risk prediction in type 2 diabetes (T2D). We assessed the long term predictive value of 4 novel CV risk markers for major CV events and mortality.

**Methods:**

We included patients with T2D who had cytokines (interleukin [IL]-6 and activin A [actA]), a maximum stress ECG test (evaluated by the normalization pattern in early recovery phase) and echocardiography (evaluated by a measure of the left ventricular filling pressure - E/Em) assessed at baseline. The primary endpoint was time to first of any of the following events: myocardial infarction, stroke, hospitalization for unstable angina pectoris and death. All outcomes were adjudicated by independent experts. We used Cox proportional hazard modeling, Harrell C-statistic and the net reclassification improvement (NRI) to assess the additional value beyond conventional markers (age, gender, prior CV disease, HDL, creatinine, diastolic BP, microalbuminuria).

**Results:**

At baseline the study cohort (n = 135, mean age/diabetes duration/HbA1c: 59 yrs/7 yrs/7.6% [59 mmol/mol], 26% females) had moderate elevated CV risk (42% microalbuminuria, mean Framingham 10 year CV-risk 9.6%). During 8.6 yrs/1153.7 person years, 26 patients experienced 36 events. All 4 novel risk markers were significantly associated with increased risk of the primary endpoint, however, only IL-6 and actA improved C-statistic and NRI (+0.119/43.2%, +0.065/20.3% respectively) compared with the conventional CV risk factors.

**Conclusions:**

IL-6 and actA may provide prognostic information on CV events and mortality in T2D beyond conventional CV risk factors.

**Trial registration:**

ClinicalTrials.gov: NCT00133718

## Introduction

Individuals with type 2 diabetes are at high risk for developing cardiovascular disease (CVD), such as coronary artery disease (CAD) and stroke, and CVD is recognized as the leading cause of premature death in people with type 2 diabetes [1]. Although state-of-the-art follow-up, including primary and secondary prevention, has reduced the cardiovascular (CV) incidence in individuals with type 2 diabetes, considerable residual CV risk remains. Therefore, together with increasing longevity, the CV burden for societies remains substantial [2]. One particular challenge in diabetes is that detection of CVD, in particular of CAD, is difficult due to atypical symptoms as well as a low sensitivity inherent in traditional non-invasive tests such as exercise ECG test or scintigraphy [3]. Moreover, although certain biomarkers have held the promise to improve CV risk prediction in people with type 2 diabetes, their applicability and implementation currently are not widespread. Novel, preferable, non-invasive tests (functional or structural) and markers (blood or urine) are therefore needed to enable earlier identification of individuals with type 2 diabetes at high risk for CVD.

Exercise ECG is frequently used to diagnose CAD in subjects with type 2 diabetes, however with lower sensitivity than in non-diabetic subjects. An evaluation of the early recovery phase of an ECG stress-test may potentially provide insight into the interplay between physiology and pathology [4,5] and our group has previously shown that detection of a pathological normalization of this phase improves sensitivity of an ECG stress test [6]. Based on these data, we hypothesized that detection of a pathological recovery phase would improve prediction of adverse outcome in people with type 2 diabetes. Another functional parameter, readily available from a conventional echocardiography examination, is the ratio E/Em which describes the ratio between early diastolic transmitral flow velocity and early diastolic mitral annular velocity. E/Em is a sensitive, non-invasive measure of the left ventricular end diastolic filling pressure in selected populations [7] and has been found to be independently associated with mortality and CV events in several disease condititions [8-10]. Since patients with type 2 diabetes are prone to develop left ventricular diastolic dysfunction, which often evolves over several years before symptoms occur [11], we hypothezised that E/Em could provide prognostic information also in this population.

In addition to novel approaches in ECG- and echocardiography assessment, soluble biomarkers have received much attention in CVD [12-15]. A large mendelian randomization analysis [15] found evidence for a causal role of IL-6 receptor signalling in the development of CAD, and proposed blockage of the IL-6 receptor as a novel therapeutic approach to prevent CAD. Our group has previously shown that interleukin (IL)-6 and activin A, a member of the transforming growth factor β superfamily, are related to the degree of CAD in patients with type 2 diabetes [16,17].

In the present study, in individuals with type 2 diabetes, we explored the association of these novel CV risk markers (pathological recovery phase, E/Em, IL-6 and activin A) with major CV events and mortality.

## Research design and methods

### Study population and study design

The present longitudinal, cohort study is part of the prospective study Asker and Bærum Cardiovascular Diabetes (ABCD) study (clinical trials.gov id: NCT00133718) that was initiated in 2002. In short, 135 patients with type 2 diabetes were recruited over 2 years (2002–2004) and underwent a comprehensive diagnostic work-up at baseline (BL) including medical history and physical examination, laboratory assessment including markers of inflammation, renal function and lipids, urinary assessment of albumin excretion, ECG, cardiopulmonary exercise test (CPET) with special emphasis on the recovery phase (ST/HR-recovery loop evaluation), echocardiography including tissue Doppler Imaging (TDI), and invasive coronary angiography.

Our primary composite endpoint was time to death or first CV event (myocardial infarction [MI], stroke or hospitalization for unstable angina pectoris [UAP]). All clinical events were adjudicated by an independent endpoint committee. During the first two years of the study, 120 of the 135 patients participated in a two year program comparing usual care provided by general practitioners to intensive structured care at the diabetic out-patient clinic. At the end of the 2 year program, all study participants were transferred back to pre-inclusion care and recommended follow-up according to guidelines.

Informed consent was obtained from all participants. The study was conducted in accordance with the Helsinki declaration and good clinical practice and was approved by the Regional Ethical Committee.

### Biochemical analyses in blood

Routine laboratory parameters were assessed in peripheral venous blood drawn in the morning after an overnight fast and immediately analyzed by the local laboratory (described in details previously [16]). Plasma levels of IL-6 and serum levels of activin A were analyzed by immunoassays from R&D Systems (Minneapolis, Minn., USA) and Serotec (Oxford, UK) as previously described [16,17]. Serum levels of high sensitivity C-reactive protein (hsCRP) were analyzed by a high-sensitivity, particle-enhanced, immunoturbidimetric assay on a modulator platform (Roche Diagnostica, Basel, Switzerland). For all assays, the intra-and interassay coefficients of variation were < 10%.

### Urinary samples

Urinary albumin excretion was determined in timed overnights urine samples. Micro-albuminuria was defined as urinary excretion > 20 μg/min in two out of three samples [18].

### Exercise testing

Exercise capacity was assessed by a modified conventional maximum symptom-limited cardiopulmonary exercise test on a cycle ergometer as described previously [16]. The ST/heart rate (HR)-slope was computer calculated using linear regression of all ST-amplitude and HR-data (obtained every 10^th^ second) during the last 4 minutes of exercise. The computer generated ST/HR- recovery loop was then evaluated in the same lead and semi-quantitatively classified to be counterclockwise (normal) or clockwise or figure 8 loop (pathological).

### Coronary angiography

Coronary angiography was performed in 91 patients according to standard procedures irrespective of results from non-invasive tests [16]. Significant CAD was defined as the presence of ≥ 50% luminal diameter narrowing of one or more of the epicardial arteries or its major branches.

### Echocardiography

Transthoracic echocardiography was obtained in 100 consenting patients by experienced research echocardiographers following guidelines for acquisition and interpretation of data [19,20] using GE Vingmed Ultrasound’s Vivid 5 or 7 with a 2.5 MHz probe (Horten, Norway). Pulsed wave Doppler mitral flow velocities were recorded in the apical 4-chamber view with the sample volume between the leaflet tips. In the same view, color-coded Tissue Doppler Imaging (TDI) loops were recorded and stored digitally and the early diastolic mitral annular velocity (Em) was measured off-line in the basal interventricular septum (EchoPac, GE Vingmed Ultrasound AS).

### Statistics

Based on the STENO-2 study (~ 715 patient years of follow-up) [21], we targeted at least 1000 patient years of follow-up to ensure that the predictive properties of the novel risk factors and tests could be assessed with a reasonable power. We used the two-sample T test to compare continuous variables. Microalbuminuria, activin A and hsCRP were log-transformed due to their skewed distributions. The Pearson chi-square test and the Fisher mid-p test were used to compare dichotomous variables [22].

We explored the relation of biomarkers to outcome with Cox proportional hazards models. The following variables were considered for inclusion in adjusted models: age, diabetes duration, gender, known history of CVD i.e., CAD, stroke or peripheral artery disease, at inclusion, allocation to treatment group first 2 study years (see methods), systolic blood pressure (BP), diastolic BP, HDL cholesterol, LDL cholesterol, HbA1c, creatinine, body mass index (BMI), smoking, microalbuminuria, left ventricle internal diastolic diameter, end diastolic volume index, and LV ejection fraction (LVEF)). Variables with p > 0.25 in univariable analyses were excluded from the final adjusted models. We assessed the proportional hazards assumption by a test based on Schoenfeld’s residuals.

To explore the additional predictive value of the new risk markers, we used Harrell’s C-statistic and the Net Reclassification Improvement (NRI) [23]. Three risk categories (defined by 5% and 10% cut-offs of estimated probabilities from logistic regression models) were used to calculate the NRI-values. We used SPSS version 18.0 (SPSS Inc., Chicago, IL) and STATA version 12 (StataCorp, College Station, TX) for statistical analysis. In all analyses, p < 0.05 was considered significant.

## Results

The observational period, which started in 2002, ended May 29^th^ 2012 and of the 135 patients that were enrolled and comprehensively assessed at BL, three patients (2.2%) were lost to follow-up, rendering 132 patients with information on outcome and vital status at study end. In two cases, cause of death was lacking in the medical records, and information was obtained from the Norwegian National Death Registry. During a follow-up time of 8.6 ± 2.1 years corresponding to 1153 person years, 26 patients (19.3%) experienced a total of 36 events (8 MIs, 8 strokes, 3 hospitalizations for UAP, 5 CV deaths and 12 non-CV deaths [3 cancers, 2 infections, 1 Parkinson’s Disease, 1 acute renal failure, 1 opioid overdose and 1 gangrenous colon due to embolies]) translating to an event rate of 3.12 and a crude mortality rate of 1.47 pr 100 person years.

**Figure 1:**
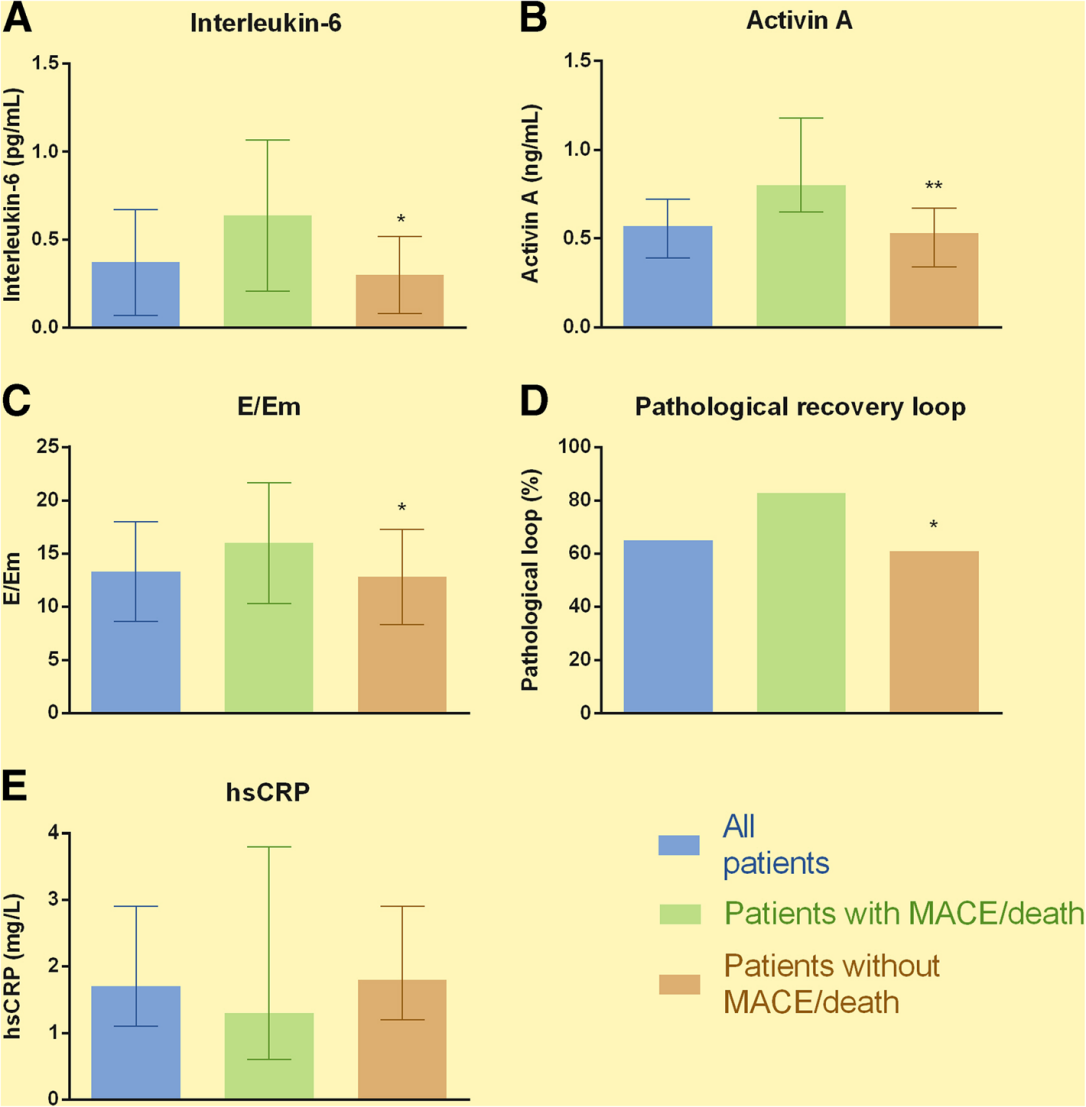
**A**-**E: Novel cardiovascular risk markers stratified by outcome.** The columns represent the mean ± SD [IL-6 and E/Em] or median (IQ range) [activin A and hsCRP] levels of novel CV markers, or proportions of participants with pathological loop [pathological loop] in all participants, in those who experienced a MACE or died, and in those who stayed event-free. *: p < 0.05, **: p < 0.01 event vs no event. SD = standard deviation, IL-6 = Interleukin-6, hsCRP = high sensitivity C-reactive protein, CV = cardiovascular, MACE = major adverse cardiovascular event.

Baseline characteristics stratified by outcome are given in Table 1 and Figure 1A-E. Patients who experienced an event were significantly older, had higher proportions of 2-or 3-vessel CAD, had more often known CAD or CVD at inclusion, had higher serum level of creatinine, lower LVEF and a higher Framingham risk score. In addition, these patients more often had a pathological recovery-loop as well as higher levels of IL-6, activin A and E/Em. In contrast, there were no differences in hsCRP, lipid parameters, HbA1c and microalbuminuria between these two groups of type 2 diabetes patients (Table 1, Figure 1A-E).

**Table 1:** Demographics, traditional CV risk factors, comorbidities and the use of medication at baseline, stratified by outcome

	**All (n = 135)**	**Patients with event (n = 26)**	**Patients without event (n = 109)**	**P value**
**Conventional CV risk markers**				
Age (yrs)	58.5 ± 10.0	64.2 ± 8.6	57.2 ± 9.9	0.001
Females n (%)	35 (26)	29 (27)	6 (23)	0.81
Diabetes duration (yrs)	6.5 ± 6.3	5.6 ± 4.8	6.6 ± 6.4	0.46
BMI (kg/m^2^)	30.0 ± 5.4	29.7 ± 6.2	30.1 ± 5.2	0.75
Systolic BP (mmHg)	142 ± 20	144 ± 21	142 ± 20	0.60
Diastolic BP (mmHg)	83 ± 10	83 ± 13	83 ± 9	0.81
HbA1c (%/mmol/mol)	7.6 ± 1.6/59.3 ± 17.0	7.5 ± 1.6/58.3 ± 17.0	7.6 ± 1.6/59.6 ± 17.1	0.74
FBG (mmol/L)	9.5 ± 3.2	9.4 ± 3.2	9.6 ± 3.3	0.80
Total cholesterol (mmol/L)	4.9 ± 1.0	4.8 ± 1.4	5.0 ± 1.0	0.47
LDL cholesterol (mmol/L)	2.8 ± 0.9	2.7 ± 0.8	2.9 ± 0.9	0.23
HDL cholesterol (mmol/L)	1.3 ± 0.4	1.2 ± 0.3	1.3 ± 0.4	0.29
Triglycerides (mmol/L)	1.9 ± 1.8	2.2 ± 3.2	1.8 ± 1.2	0.56
Creatinine (μmol/L)	78.5 ± 15.3	86.7 ± 21.1	76.5 ± 12.9	0.025
Albuminuria (μg/min)	16.0 (9.0, 30.5)	22.0 (9.8, 39.0)	15.0 (9.0, 28.3)	0.091
Microalbuminuria n (%)	56 (42)	14 (54)	42 (39)	0.17
LVEF (%)	63 ± 8	59 ± 8	64 ± 8	0.029
Daily smoking n (%)	16 (12)	3 (12)	13 (12)	0.87
Known CAD at inclusion n (%)	16 (12)	8 (31)	8 (7)	0.002
Known CVD at inclusion n (%)*	22 (16)	10 (39)	12 (11)	0.001
Framingham 10 years risk of CHD (%)	9.6 ± 6.6	12.7 ± 6.9	8.9 ± 6.4	0.022
**Medications**				
Numbers of Blood glucose lowering medications	1.2 ± 0.9	1.4 ± 0.9	1.2 ± 0.9	0.29
Numbers of BP-lowering medications†	0.9 ± 1.0	1.1 ± 1.2	0.9 ± 1.0	0.39
On statins n (%)	61 (45)	14 (54)	47 (50)	0.38
On ASA n (%)	45 (33)	12 (46)	33 (30)	0.16

Data given as means ± SD, medians (interquartile range) or proportions.

*Abbreviations*: *CV* Cardiovascular, *BMI* Body mass index, *BP* Blood Pressure, *FBG* Fasting blood glucose, *LVEF* Left ventricular ejection fraction, *CAD* Coronary artery disease, *CVD* Cardiovascular disease, *CHD* Coronary heart disease, *ACEI* Angiotensin-converting enzyme inhibitor, *ARB* Angiotensin II receptor blocker, *ASA* Acetylsalicylic acid.

* = known history of CAD, stroke or peripheral artery disease at inclusion.

† = ACEI/ARBs/beta blockers/Ca-blockers/diuretics/combined therapy.

**Figure 2:**
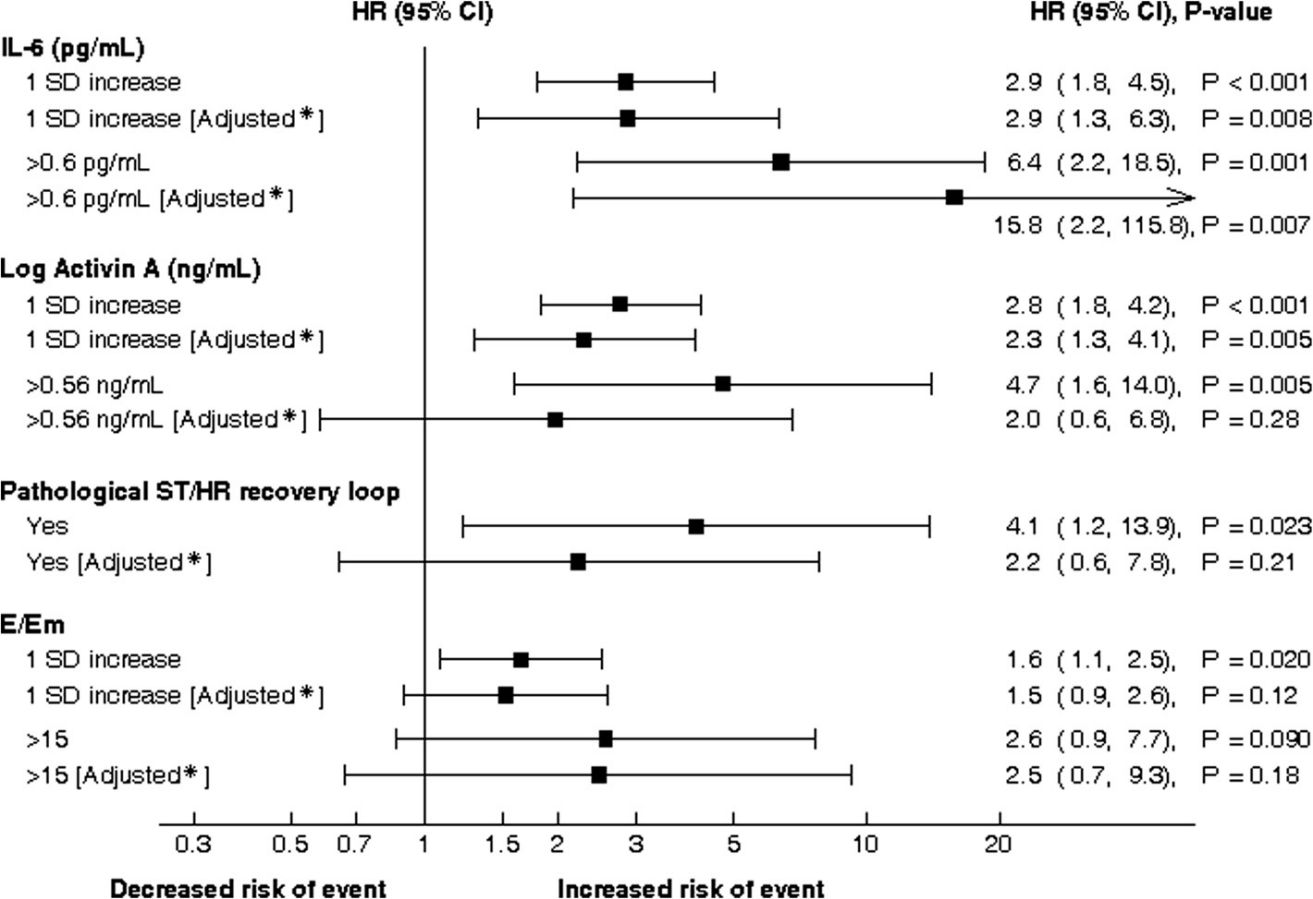
**Unadjusted and adjusted hazard ratios for major CV events and death for the novel CV risk markers.** * adjusted for age, gender, known CVD at inclusion, diastolic blood pressure, s-HDL-cholesterol, log microalbuminuria and s-creatinine. Abbreviations: CV = cardiovascular, IL-6 = Interleukin-6, SD = Standard Deviation.

In a univariate Cox regression analysis, IL-6, activin A, E/Em and pathological recovery-loop, but not CRP (data not shown), were all significantly associated with the outcome (Figure 2). Several conventional risk factors were also significantly associated with outcome in univariate analysis: age (HR = 1.08, 95% CI 1.03-1.13), known history of CVD at inclusion (HR = 3.53, 95% CI 1.58-7.87) and creatinine (HR = 1.41, 95% CI 1.09-1.82).

Activin A and IL-6 (also when dichotomized at median values, respectively IL-6 > 0.6 pg/mL and activin A > 0.56 ng/mL), but not E/Em and pathological recovery loop maintained their predictive value when adjusted for the conventional risk factors (age, gender, known CV disease, diastolic blood pressure, microalbuminuria and serum levels of HDL-cholesterol and creatinine) in multivariate analysis (Figure 2). Table 2 shows C-statistics and NRI for our conventional risk factors (clinical and biochemical) and different combinations of novel biomarkers on top of this standard model. Two of the explored novel markers (i.e., IL-6 and activin A) significantly improved risk classification in this study cohort and fitted overall with the results from the Cox regression. Moreover, when combining these two markers, we observed a 47% improvement in risk classification.

**Table 2:** C-statistics and net reclassification improvement (NRI) for the conventional risk markers alone, and in combination with one or more novel risk marker

	**Harrell’s C**	**NRI, P-value**
Standard (STD) model alone	0.794	Reference model
STD + IL-6	0.913	43.2%, p = 0.001
STD + log ActivinA	0.859	20.3%, p = 0.013
STD + IL-6 + log ActivinA	0.923	46.5%, p < 0.001
STD + E/Em + pathol recovery loop	0.891	21.5%, p = 0.099

The NRI calculations are based on estimated probabilities of event from logistic regression models. The three risk categories used are defined by the probability cut-offs 5% and 10%.

STD model: age, gender, known CVD, diastolic blood pressure, microalbuminuria and serum levels of HDL-cholesterol and creatinine

*Abbreviations*: *STD* Standard, *IL-6* Interleukin-6, *pathol* Pathological.

## Discussion

Our main findings in the present study were that the novel risk markers and tests IL-6, activin A, pathological recovery loop and E/Em all were significantly associated with a combined endpoint of CV events and mortality in a cohort of patients with type 2 diabetes at moderate CV risk. For IL-6 and activin A, the association to CV events and mortality was kept when adjusting for traditional CV risk factors. Also, when added to a model based on traditional risk factors, IL-6 and activin A, but not E/Em or post-exercise recovery loop, improved risk classification beyond conventional CV risk factors as assessed by C-statistics and NRI.

Of interest is that IL-6 has emerged as an important cytokine with a link to atherosclerosis and diabetes [24,25], and evidence from two large genetic studies indicates causality between IL-6 receptor signalling and CAD [15,26]. Supportive to our results are the results by Martin-Cordero et al. of increased levels of IL-6 in high CV risk rats (genetically obese rats with metabolic syndrome) [27], and the study by Zhou et al. which found increased levels of IL-6 in epicardial tissue in humans with CAD [28], highlighting this association. Further, longitudinal studies in healthy post-menopausal women [29], healthy men [30] and elderly [31] have shown the ability of IL-6 to be a predictor of future CV events and mortality. However, data evaluating IL-6’s long term ability to predict clinical outcomes in people with type 2 diabetes is scarce. In a relatively small cross-sectional study on men with type 2 diabetes, comparison of different CV risk markers in patients with microvascular complications with patients with established CAD [32], revealed no significant difference in IL-6 levels. A German group investigated the association between IL-6 and primary CV events in 1072 patients with type 2 diabetes during 5 years follow-up and found a significant association with adverse events [33]. Our study further indicates an association between IL-6 and CV events and all cause mortality in people with type 2 diabetes potentially giving prognostic information beyond traditional risk factors.

Activin A exhibits both anti- and pro inflammatory effects [34] and has been implicated in the pathogenesis of diabetes. It has also been related to CAD, potentially reflecting a counteracting mechanism [35], and may reflect disease severity of type 2 diabetes [17,36,37]. The present study further supports a role for activin A in diabetes and related CV events and death by showing an independent association between activin A and adverse outcome in type 2 diabetes.

CRP is regarded as a reliable down-stream marker of inflammation, and although IL-6 is suggested to be one of its main inducers, we could not find any association of CRP with CV events and death in this population with type 2 diabetes. The reason for this finding is at present not clear, but underscores that it is unlikely that one marker could reflect all inflammatory pathways involved in complex disorders like type 2 diabetes and its CV complications. Moreover, although IL-6 is an up-stream activator of CRP synthesis, this pleiotrophic cytokine also has a wide range of other effects not related to induction of acute phase proteins.

E/Em is a reliable predictor of major CV events and death in chronic renal failure, ischemic heart disease and in hypertrophic cardiomyopathy [8,9,38]. Our data indicated that E/Em, an easily attainable ratio in those patients that undergo echocardiography, is associated with major CV events and death also in people with type 2 diabetes. We did, however, not find a clear improvement in the prediction of outcome when adding E/Em to a model consisting of conventional CV risk factors, potentially at least partly reflecting a relatively low number of patients.

Likewise, although we found that pathological recovery loop was significantly associated to outcome, it did not improve prediction of this beyond standard risk factor assessment.

### Limitations

Our study has some limitations including a potential low generalizability since all patients were Caucasians and examined at the same center. The number of participants was relatively low and not all had IL-6 and E/Em measured, which led to an even more reduced number of events that could be related to these markers and tests. Our study is also insufficiently powered to analyze the individual components of the composite endpoint in fatal and non-fatal CV events. Moreover, less than 1/3 of the first events had cardiac origin; the remaining being strokes, stroke related deaths, or death from non CV causes.

The strength of our study is foremost the long observation-time and accurate follow-up data on outcome, and that few patients were lost to follow-up.

## Conclusion

Circulating levels of IL-6 and activin A may provide prognostic information on CV events and mortality beyond traditional CV risk factors. As these both are easily implementable in clinical practice they could improve CV prevention strategies in people with type 2 diabetes. We suggest that these parameters as well as E/Em and pathological recovery loop should be further validated in other and larger cohorts of patients with type 2 diabetes.

## Abbreviations

MACE: Major adverse cardiovascular event; CV: Cardiovascular; CVD: Cardiovascular disease; CAD: Coronary artery disease; IL: Interleukin; CPET: Cardiopulmonary exercise test; TDI: Tissue doppler imaging; MI: Myocardial infarction; UAP: Unstable angina pectoris; hsCRP: High sensitivity C-reactive protein; ST/HR: ST/heart rate; LV: Left ventricular; LVEF: Left ventricular ejection fraction; BMI: Body mass index; NRI: Net reclassification improvement.

## Competing interests

Odd Erik Johansen is employed by Boehringer Ingelheim. Kåre I. Birkeland has served as a consultant for or received lecture fees or travel reimbursement from Novo Nordisk, Eli Lilly, Sanofi-Aventis, MSD, Boehringer Ingelheim, Bristol-Myers Squibb, Novartis or Pfizer.

Lars Gullestad has received lecture fees from Eli Lilly. All other authors declare no competing interests.

## Authors’ contributions

OEJ, KIB and LG. designed the study. APO, EO, OEJ, SA, KE, TU and PA collected and researched the data. APO coordinated the writing of the manuscript. MWF was the statistical expert. All authors contributed to discussion, reviewed the manuscript, and approved the final work. APO and OEJ are the guarantors of this work and, as such, had full access to all the data in the study and take responsibility for the integrity of the data and the accuracy of the data analysis.

## References

[B1] Vaccaro O Eberly LE Neaton JD Yang L Riccardi G Stamler J Impact of diabetes and previous myocardial infarction on long-term survival: 25-year mortality follow-up of primary screenees of the Multiple Risk Factor Intervention Trial Arch Intern Med 2004 164 13 1438 1443 10.1001/archinte.164.13.1438 15249353

[B2] Sloan FA Bethel MA Ruiz D Jr Shea AM Feinglos MN The growing burden of diabetes mellitus in the US elderly population Arch Intern Med 2008 168 2 192 199 discussion 199 10.1001/archinternmed.2007.35 18227367

[B3] Lee DP Fearon WF Froelicher VF Clinical utility of the exercise ECG in patients with diabetes and chest pain Chest 2001 119 5 1576 1581 10.1378/chest.119.5.1576 11348969

[B4] Kligfield P Ameisen O Okin PM Heart rate adjustment of ST segment depression for improved detection of coronary artery disease Circulation 1989 79 2 245 255 10.1161/01.CIR.79.2.245 2644054

[B5] Kligfield P Principles of simple heart rate adjustment of ST segment depression during exercise electrocardiography Cardiol J 2008 15 2 194 200 18651407

[B6] Johansen OE Bjuro T Endresen K Blaasaas KG Birkeland K Aakhus S Gullestad L Heart rate adjustments and analysis of recovery patterns of ST-segment depression in type 2 diabetes Int J Cardiol 2008 127 1 129 132 10.1016/j.ijcard.2007.04.022 17532067

[B7] Ommen SR Nishimura RA Appleton CP Miller FA Oh JK Redfield MM Tajik AJ Clinical utility of Doppler echocardiography and tissue Doppler imaging in the estimation of left ventricular filling pressures: A comparative simultaneous Doppler-catheterization study Circulation 2000 102 15 1788 1794 10.1161/01.CIR.102.15.1788 11023933

[B8] Wang AY Wang M Lam CW Chan IH Zhang Y Sanderson JE Left ventricular filling pressure by Doppler echocardiography in patients with end-stage renal disease Hypertension 2008 52 1 107 114 10.1161/HYPERTENSIONAHA.108.112334 18474835

[B9] Hillis GS Moller JE Pellikka PA Gersh BJ Wright RS Ommen SR Reeder GS Oh JK Noninvasive estimation of left ventricular filling pressure by E/e' is a powerful predictor of survival after acute myocardial infarction J Am Coll Cardiol 2004 43 3 360 367 10.1016/j.jacc.2003.07.044 15013115

[B10] Kitaoka H Kubo T Hayashi K Yamasaki N Matsumura Y Furuno T Doi YL Tissue Doppler imaging and prognosis in asymptomatic or mildly symptomatic patients with hypertrophic cardiomyopathy Eur Heart J Cardiovasc Imaging 2013 14 6 544 549 10.1093/ehjci/jes200 23060455

[B11] From AM Scott CG Chen HH The development of heart failure in patients with diabetes mellitus and pre-clinical diastolic dysfunction a population-based study J Am Coll Cardiol 2010 55 4 300 305 10.1016/j.jacc.2009.12.003 20117433 PMC3878075

[B12] Phillips DJ de Kretser DM Hedger MP Activin and related proteins in inflammation: not just interested bystanders Cytokine Growth Factor Rev 2009 20 2 153 164 10.1016/j.cytogfr.2009.02.007 19261538

[B13] Naka T Nishimoto N Kishimoto T The paradigm of IL-6: from basic science to medicine Arthritis Res 2002 4 Suppl 3 S233 242 10.1186/ar565 12110143 PMC3240141

[B14] Danesh J Kaptoge S Mann AG Sarwar N Wood A Angleman SB Wensley F Higgins JP Lennon L Eiriksdottir G Long-term interleukin-6 levels and subsequent risk of coronary heart disease: two new prospective studies and a systematic review PLoS Med 2008 5 4 e78 10.1371/journal.pmed.0050078 18399716 PMC2288623

[B15] Hingorani AD Casas JP The interleukin-6 receptor as a target for prevention of coronary heart disease: a mendelian randomisation analysis Lancet 2012 379 9822 1214 1224 22421340 10.1016/S0140-6736(12)60110-X PMC3316968

[B16] Johansen OE Birkeland KI Orvik E Flesland O Wergeland R Ueland T Smith C Endresen K Aukrust P Gullestad L Inflammation and coronary angiography in asymptomatic type 2 diabetic subjects Scand J Clin Lab Invest 2007 67 3 306 316 10.1080/00365510601045088 17454845

[B17] Ueland T Aukrust P Aakhus S Smith C Endresen K Birkeland KI Gullestad L Johansen OE Activin A and cardiovascular disease in type 2 diabetes mellitus Diab Vasc Dis Res 2012 9 3 234 237 10.1177/1479164111431171 22234949

[B18] Executive summary: standards of medical care in diabetes—2013 Diabetes Care 2013 36 Supplement 1 S4 S10 23264424 10.2337/dc13-S004 PMC3537272

[B19] Gottdiener JS Bednarz J Devereux R Gardin J Klein A Manning WJ Morehead A Kitzman D Oh J Quinones M American Society of Echocardiography recommendations for use of echocardiography in clinical trials J Am Soc Echocardiogr 2004 17 10 1086 1119 15452478 10.1016/j.echo.2004.07.013

[B20] Lang RM Bierig M Devereux RB Flachskampf FA Foster E Pellikka PA Picard MH Roman MJ Seward J Shanewise JS Recommendations for chamber quantification: a report from the American Society of Echocardiography's Guidelines and Standards Committee and the Chamber Quantification Writing Group, developed in conjunction with the European Association of Echocardiography, a branch of the European Society of Cardiology J Am Soc Echocardiogr 2005 18 12 1440 1463 10.1016/j.echo.2005.10.005 16376782

[B21] Gaede P Lund-Andersen H Parving HH Pedersen O Effect of a multifactorial intervention on mortality in type 2 diabetes N Engl J Med 2008 358 6 580 591 10.1056/NEJMoa0706245 18256393

[B22] Lydersen S Fagerland MW Laake P Recommended tests for association in 2 × 2 tables Stat Med 2009 28 7 1159 1175 10.1002/sim.3531 19170020

[B23] Pencina MJ D'Agostino RB Sr D'Agostino RB Jr Vasan RS Evaluating the added predictive ability of a new marker: from area under the ROC curve to reclassification and beyond Stat Med 2008 27 2 157 172 discussion 207–112 10.1002/sim.2929 17569110

[B24] Spranger J Kroke A Mohlig M Hoffmann K Bergmann MM Ristow M Boeing H Pfeiffer AF Inflammatory cytokines and the risk to develop type 2 diabetes: results of the prospective population-based European Prospective Investigation into Cancer and Nutrition (EPIC)-Potsdam Study Diabetes 2003 52 3 812 817 10.2337/diabetes.52.3.812 12606524

[B25] Koenig W Khuseyinova N Biomarkers of atherosclerotic plaque instability and rupture Arterioscler Thromb Vasc Biol 2007 27 1 15 26 10.1161/01.ATV.0000251503.35795.4f 17082488

[B26] Sarwar N Butterworth AS Freitag DF Gregson J Willeit P Gorman DN Gao P Saleheen D Rendon A Nelson CP Interleukin-6 receptor pathways in coronary heart disease: a collaborative meta-analysis of 82 studies Lancet 2012 379 9822 1205 1213 22421339 10.1016/S0140-6736(11)61931-4 PMC3316940

[B27] Martin-Cordero L Garcia JJ Hinchado MD Ortega E The interleukin-6 and noradrenaline mediated inflammation-stress feedback mechanism is dysregulated in metabolic syndrome: effect of exercise Cardiovasc Diabetol 2011 10 42 10.1186/1475-2840-10-42 21599899 PMC3118326

[B28] Zhou Y Wei Y Wang L Wang X Du X Sun Z Dong N Chen X Decreased adiponectin and increased inflammation expression in epicardial adipose tissue in coronary artery disease Cardiovasc Diabetol 2011 10 1 2 10.1186/1475-2840-10-2 21226932 PMC3032658

[B29] Ridker PM Hennekens CH Buring JE Rifai N C-reactive protein and other markers of inflammation in the prediction of cardiovascular disease in women N Engl J Med 2000 342 12 836 843 10.1056/NEJM200003233421202 10733371

[B30] Ridker PM Rifai N Stampfer MJ Hennekens CH Plasma concentration of interleukin-6 and the risk of future myocardial infarction among apparently healthy men Circulation 2000 101 15 1767 1772 10.1161/01.CIR.101.15.1767 10769275

[B31] Harris TB Ferrucci L Tracy RP Corti MC Wacholder S Ettinger WH Jr Heimovitz H Cohen HJ Wallace R Associations of elevated interleukin-6 and C-reactive protein levels with mortality in the elderly Am J Med 1999 106 5 506 512 10.1016/S0002-9343(99)00066-2 10335721

[B32] Davenport C Ashley DT O'Sullivan EP Corley BT Fitzgerald P Agha A Thompson CJ O'Gorman DJ Smith D Identifying coronary artery disease in men with type 2 diabetes: osteoprotegerin, pulse wave velocity, and other biomarkers of cardiovascular risk J Hypertens 2011 29 12 2469 2475 10.1097/HJH.0b013e32834c1e95 21970938

[B33] Herder C Schottker B Rothenbacher D Roden M Kolb H Muller H Brenner H Interleukin-6 in the prediction of primary cardiovascular events in diabetes patients: results from the ESTHER study Atherosclerosis 2011 216 1 244 247 10.1016/j.atherosclerosis.2011.01.041 21334625

[B34] Sideras P Apostolou E Stavropoulos A Sountoulidis A Gavriil A Apostolidou A Andreakos E Activin, neutrophils, and inflammation: just coincidence? Semin Immunopathol 2013 35 4 481 99 10.1007/s00281-013-0365-9 23385857 PMC7101603

[B35] Andersen GO Ueland T Knudsen EC Scholz H Yndestad A Sahraoui A Smith C Lekva T Otterdal K Halvorsen B Activin A levels are associated with abnormal glucose regulation in patients with myocardial infarction: potential counteracting effects of activin A on inflammation Diabetes 2011 60 5 1544 1551 10.2337/db10-1493 21464440 PMC3292329

[B36] Wu H Wu M Chen Y Allan CA Phillips DJ Hedger MP Correlation between Blood Activin Levels and Clinical Parameters of Type 2 Diabetes Exp Diabetes Res 2012 2012 410579 23304117 10.1155/2012/410579 PMC3533484

[B37] Weigert J Neumeier M Wanninger J Schober F Sporrer D Weber M Schramm A Wurm S Stogbauer F Filarsky M Adiponectin upregulates monocytic activin A but systemic levels are not altered in obesity or type 2 diabetes Cytokine 2009 45 2 86 91 10.1016/j.cyto.2008.10.017 19128983

[B38] Kitaoka H Kubo T Hayashi K Yamasaki N Matsumura Y Furuno T Doi YL Tissue Doppler imaging and prognosis in asymptomatic or mildly symptomatic patients with hypertrophic cardiomyopathy Eur Heart J Cardiovasc Imaging 2012 14 6 544 9 23060455 10.1093/ehjci/jes200

